# Development of a Novel Inhalational Model of Invasive Pulmonary Aspergillosis in Rats and Comparative Evaluation of Three Biomarkers for Its Diagnosis

**DOI:** 10.1371/journal.pone.0100524

**Published:** 2014-06-23

**Authors:** Suhail Ahmad, Ali A. Al-Shaikh, Ziauddin Khan

**Affiliations:** Department of Microbiology, Faculty of Medicine, Kuwait University, Safat, Kuwait; David Geffen School of Medicine at University of California Los Angeles, United States of America

## Abstract

*Aspergillus fumigatus*, a thermotolerant fungus, is the main causative agent of invasive pulmonary aspergillosis (IPA) in immunocompromised patients that is associated with high mortality rates. Early diagnosis of IPA is crucial for mortality reduction and improved prognosis. An experimental inhalational model of IPA was developed in rats and the efficacy of three biomarkers, namely β-D-glucan (BDG), a panfungal marker, galactomannan (GM), a genus-specific marker, and *A. fumigatus* DNA, a species-specific marker was evaluated in serum and bronchoalveolar lavage (BAL) specimens at different time points postinfection for early diagnosis of IPA. BDG and GM were detected by using commercial Fungitell and Platelia *Aspergillus* EIA kits, respectively. *A. fumigatus* DNA was detected by developing a sensitive, single-step PCR assay. IPA was successfully developed in immunosuppressed rats and all animals until 5 days post-infection were positive for *A. fumigatus* by culture and KOH-calcofluor microscopy also showed *A. fumigatus* in 19 of 24 (79%) lung tissue samples. Fourteen of 30 (47%) and 27 of 30 (90%) serum and BAL specimens, respectively, were positive for all three biomarkers with 100% specificity (none of sera or BAL specimens of 12 control rats was positive for biomarkers). Our data show that BAL is a superior specimen than serum and combined detection of BDG, GM and *A. fumigatus* DNA provide a sensitive diagnosis of IPA in an experimental animal model. Moreover, combined detection of GM and DNA in BAL and detection of either GM or DNA in serum was also positive in 27 of 30 (90%) animals. For economic reasons and considering that the positive predictive value of BDG is low, the detection of GM and/or DNA in serum and BAL samples has the potential to serve as an integral component of the diagnostic-driven strategy in high-risk patients suspected for IPA.

## Introduction

Invasive pulmonary aspergillosis (IPA) is a serious infection in patients with prolonged neutropenia, transplant recipients and other severely immunocompromised patients. *Aspergillus fumigatus*, one of the most prevalent airborne fungal pathogen, is the principal etiological agent implicated in IPA [1], [2]. Proper patient management and therapeutic success depends on rapid diagnosis and early initiation of appropriate antifungal therapy [3], [4]. Early diagnosis of IPA remains challenging as few diagnostic tools are available and delays in diagnosis contribute to the high mortality associated with this disease [5]. Mortality rates of IPA range from nearly 30% in some patient populations to as high as 90% in severely neutropenic patients [6]. Conventional laboratory techniques for the diagnosis of IPA include direct microscopic examination of fungal elements in clinical specimens, lung tissue histology and culture of respiratory secretions. Direct microscopy is often negative, histological confirmation is difficult to obtain due to concomitant thrombocytopenia and culture positivity even in confirmed cases of IPA typically remains less than 50% [4], [7]. Current culture-based methods for *Aspergillus* species for the evaluation of bronchoalveolar lavage (BAL) specimens also have low sensitivity as only 45 to 62% of BAL specimens from proven cases of IPA yield the fungus [8], [9]. The radiological diagnosis of IPA has also been suggested to guide clinical management of high-risk patients. The demonstration of characteristic ‘halo’ and ‘air-crescent’ signs on high-resolution computed tomography (CT) scan may suggest IPA in nearly half (50%) of neutropenic patients, however, these signs are not 100% specific [10].

The detection of biomarkers (antigens and/or DNA) in consecutive serum or, to a lesser extent, in BAL samples is an attractive strategy for early diagnosis of IPA before overt disease develops [11]. The detection of two cell wall antigens; β-D-glucan (BDG) and galactomannan (GM) is recognized by the European Organization for Research and Treatment of Cancer/Invasive Fungal Infections Cooperative Group, National Institute of Allergy and Infectious Diseases Mycoses Study Group (EORTC/MSG) as a mycological criterion for defining IPA [12]. The assays for detection of GM and BDG are commercially available and exhibit nearly similar performance (∼80% sensitivity and 85% specificity) for the diagnosis of IPA [13], [14]. However, both BDG and GM assays are susceptible to yield both, false-positive and false-negative results [11], [13], [14]. Detection of an extracellular glycoprotein antigen secreted during active growth of *Aspergillus* spp. by an immunochromatographic assay has also shown promise for the diagnosis of IPA but the method has not yet been extensively evaluated [15].

Molecular methods for the diagnosis of IPA are also being evaluated and different formats of PCR have been used as alternative or adjunct test and combined antigen and PCR testing has provided improved diagnosis compared to individual assay performance [2], [11], [16]–[19]. Some of the molecular methods require expensive probes and sophisticated equipment which are not readily available in most of the developing countries [20], [21]. Furthermore, the choice of using appropriate specimen (whole blood, serum or BAL) readily available, lack of standardization of DNA extraction and amplification protocols, differences in DNA (single or multiple copy) targets and variations in the methods used to detect amplified products (direct detection or by using expensive probes) have made it difficult to compare results between laboratories and restricted their application to specialist molecular diagnostic laboratories [11]. To overcome these difficulties, animal models of IPA have been used to develop novel methods for DNA detection and to evaluate the performance of different biomarkers for facilitating early diagnosis of IPA [22], [23]. The objective of this study was to develop and evaluate the performance of a sensitive, single-step PCR assay for detection of *A. fumigatus* DNA in serum and BAL specimens obtained from an inhalational rat model of IPA at different time points postinfection. The data were also compared with the results of culture as well as the detection of GM and BDG levels.

## Materials and Methods

### Reference strains and extraction of fungal DNA

The reference fungal strains used in the study included *A. fumigatus* (CBS 113.26), *Aspergillus flavus* (CBS 113.32), *Aspergillus terreus* (CBS 106.25*), Fusarium solani* (ATCC 36031), *Fusarium oxysporum* (CBS 109898), *Candida albicans* (ATCC 76615), *Candida parapsilosis* (ATCC 22019), *Candida dubliniensis* (CD36), *Candida glabrata* (CBS 138), *Candida tropicalis* (ATCC 750), *Cryptococcus neoformans* (CBS 7779), *Trichosporon asahii* (CBS 2479), *Trichosporon mucoides* (CBS 7625) and *Trichosporon inkin* (CBS 5585). The extraction of fungal DNA from cultures, BAL and serum samples was carried out by using phenol extraction procedure as described previously [24], [25] and detailed below.

The conidia of reference *Aspergillus* and *Fusarium* species were inoculated in 6 cm petri dishes containing 1.5 ml YPD (yeast extract 10 g, peptone 20 g, dextrose 20 g/L; pH 6.5) medium and grown at 30°C for 72 h. The mycelial mat was pulled using a pipette tip and dried on Whatman paper for 10 minutes. The mycelial mat was transferred into 50-ml polypropylene screw cap tubes containing six glass beads (4 mm diameter). The tubes were immersed in liquid nitrogen for 10 s and vortexed vigorously for 30 s. DNA extraction buffer (0.8 ml) containing 0.2 M Tris-HCl (pH 7.6), 10 mM EDTA, 0.5 M NaCl, and 1% sodium dodecyl sulfate (SDS) and 0.8 ml of phenol/chloroform/isoamylalcohol (25∶24∶1, v/v/v) was added. The contents were mixed and an aliquot of 0.7 ml was transferred to a 1.5 ml microcentrifuge tube, and centrifuged at 12,000 xg for 15 min at 4°C. The supernatant was transferred to a fresh tube and extracted with an equal volume of phenol-chloroform-isoamylalcohol. The aqueous phase was extracted once with chloroform-isoamylalcohol (24∶1, v/v). The DNA in the supernatant was precipitated with 0.6 volumes of isopropanol and centrifuged at 12,000 xg for 15 min at 4°C. The pelleted DNA was washed with 70% ethanol, dried at room temperature and dissolved in 25 µl of sterile deionized water. The DNA samples were stored at −20°C until used for PCR.

Yeast (*Candida* spp., *C. neoformans* and *Trichosporon* spp.) species were grown in 15 ml of YPD medium for 48 h at 37°C in a shaker incubator, the cells were harvested by centrifugation at 3000 xg for 101min at 4°C and washed twice using Tris-EDTA buffer (TE, 10 mM Tris-HCl, pH 7.5; 1 mM EDTA). The cells were lysed by adding 50 µg of lyticase enzyme (Sigma-Aldrich) in 500 µl of 1 M sorbitol. After 1 h at 37°C, the contents were transferred to a microcentrifuge tube and spun at 12,000 xg for 10 min at room temperature. The pellet was re-suspended in 450 µl of 3x TE buffer (30 mM Tris-HCl, pH 7.5; 3 mM EDTA) and 25 µl of 25% SDS and incubated at 65°C for 30 min. Potassium acetate (250 µl of 3 M, pH 5.4) was added and after 45 min on ice, the tubes were centrifuged at 12,000 xg for 10 min at 4°C. The supernatant (400 µl) was extracted with an equal volume of buffered phenol (Sigma-Aldrich), phenol:chloroform:isoamyl alcohol (25∶24∶1, v/v/v/) and then with chloroform:isoamyl alcohol (24∶1, v/v). The DNA in the aqueous phase was precipitated as described above and the dried pellet was dissolved in 100 µl of sterile deionized water.

The fungal DNA from serum and BAL specimens was extracted by adding 200 µl of serum or BAL sediment (re-suspended in 400 µl of deionized sterile water) to 500 µl of 6 M guanidine isothiocyanate and 500 µl of buffered phenol. The mixture was boiled for 5 min, immersed in liquid nitrogen for one min and then thawed at room temperature. The process was repeated three times and then 250 µl of chloroform/isoamylalcohol (24∶1, v/v) was added. The sample was mixed and then centrifuged at 12,000 xg for 10 min. The supernatant (500 µl) was transferred to a fresh tube, extracted with an equal volume of chloroform:isoamyl alcohol (24∶1, v/v) and then the DNA was precipitated by the addition of 0.7 volumes of isopropanol. After 30 min at −70°C, the pelleted DNA was recovered by centrifugation at 12,000 xg for 15 min at 4°C, the pellet was washed twice with 70% ethanol, dried and then re-suspended in 25 µl of 10 mM Tris HCl (pH 8.0).

### Inoculum preparation and standardization of inhalation model

**Figure 1:**
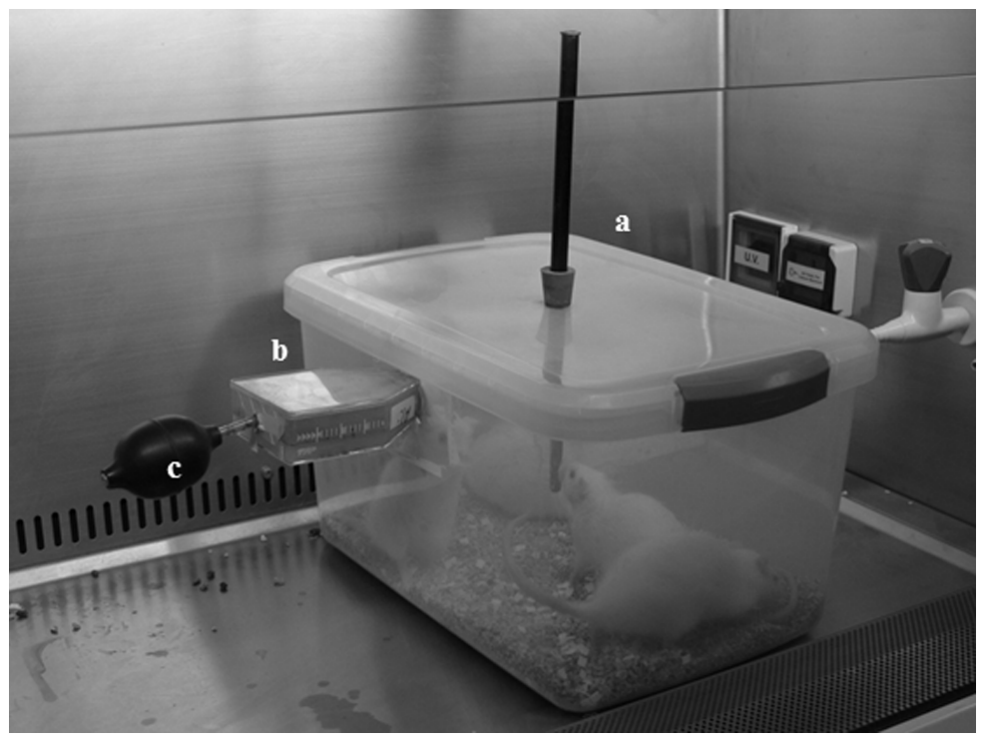
Exposure chamber used for infection of immunosuppressed rats with *A. fumigatus*. The figure shows the chamber (**a**) where the rats were kept, *A. fumigatus* culture flask (**b**) that was connected directly to the chamber and the pump (**c**) that was used to manually transfer conidia of *A. fumigatus* to the chamber for exposing immunosuppressed rats.

Female Wistar rats (18 to 25 weeks old, 180–230 g) bred and maintained in the Central Animal Facility of the Faculty of Medicine, were subjected to inhalation of *A. fumigatus* conidia. Inoculum of reference *A. fumigatus* strain (CBS 113.26) was prepared by growing the fungus on 50 ml of Sabouraud dextrose agar (SDA) medium (Oxoid Ltd.) in flat 275 ml Tissue Cell Culture bottles (Thermo Scientific Nunc) for 1 week at 37°C to ensure heavy growth [24], [25]. Just before use, a small hole was made at the other end opposite the lid by carefully using a hot metal rod in a safety cabinet and a sterile cotton-plugged tube was inserted tightly. The lid was removed and the neck was connected to the specially designed low-cost, acrylic chamber while the other cotton-plugged end was connected to a manual pump as shown in Figure 1. Cortisone acetate was chosen as the single immunosuppressing agent and the dosing (200 mg/kg body weight) and frequency of cortisone acetate administration were adopted from previously published experimental rat models of pulmonary aspergillosis [26], [27]. Each animal was immunosuppressed with intramuscular injections of cortisone acetate given on 4 and 2 days before exposure, on the day of exposure and 2 and 4 days after exposure with an intended period of one week of follow-up based on observations that the initial 4 days of IPA are critical for accurate diagnosis in both, experimental models and human subjects [28], [29]. The conidia were pumped (5 times/min) directly into the chamber housing immunosuppressed rats with complete squeezing of the palm-size manual pump connected to the culture bottles (Figure 1) for 5, 10, 15 or 20 min in a safety cabinet to avoid contamination of the facility. The exposed animals were moved to new cages after completion of exposure time, provided food and sterile water *ad libidum* and were monitored daily for mortality or were sacrificed at different time points. The appropriate dose, and exposure time were standardized for the intended period of the follow-up (7 days). The animals (four rats in each group) were subjected to inhalation of *A. fumigatus* conidia, the entire lung tissue was removed either immediately after exposure (to measure the infecting dose) or after 2, 4 or 6 days after exposure (to ascertain the presence of fungal elements in microscopic examination of lung tissue and positive lung tissue culture and survival up to 7 days) from sacrificed animals, homogenized in 1.0 ml of 1× phosphate buffered saline (PBS) and 100 µl of 10-fold serial dilutions were plated onto SDA plates for viable count. The mean colony forming units (cfu)/g lung tissue were calculated.

### Collection of specimens from experimental rats exposed to *A. fumigatus* conidia

A total of 30 immunosuppressed rats exposed to *A. fumigatus* conidia were housed individually in metabolic cages and given food pellets and water *ad libidum*. The exposed rats were sacrificed in groups of six on different days postinfection for the collection of various specimens in a safety cabinet to avoid contamination with aerial fungal spores. No cotton or gauze derivatives were used during sacrifice and collection of specimens and every effort was made to avoid BDG contamination. Freshly autoclaved scissors, forceps and freshly-opened or autoclaved plastic ware/glassware were used. Serum samples, separated from blood obtained through cardiac puncture, were used for the detection of BDG, GM, and *A. fumigatus* DNA. The BAL was collected by exposing the trachea and lavaging the lung four times with one ml of phosphate-buffered saline (PBS) in a biological safety cabinet to ensure *Aspergillus*-free environment. The sample was centrifuged at 10,000×g for 10 min. The supernatant was used for the detection of BDG and GM, while the sediment was used for culture on SDA and detection of *A. fumigatus* DNA. A portion of lung tissue was used for microscopic examination and culture on SDA plates supplemented with chloramphenicol (50 mg/L). Additionally, 12 healthy rats were also sacrificed and serum, BAL and lung tissue specimens were collected for use as controls for culture and detection of BDG, GM, and *A. fumigatus* DNA. To avoid false-positivity for the detection of GM, no antibacterial/β-lactam antibiotics were used for control or immunosuppressed animals.

### Detection of *A. fumigatus* DNA by PCR

A single-step PCR assay was developed by carefully designing two *A. fumigatus*-specific primers; AFUF (5′-CGAGTGAGGGCCCTCTGGGTCCA-3′) and AFUR (5′-ACGATAATCAACTCAGACTGCATA-3′), derived from the internally transcribed spacer (ITS)-1 region sequence of rDNA. The specificity of both the primers for *A. fumigatus* was suggested by BLAST searches and was tested by amplification of genomic DNA from other *Aspergillus* species or other common fungal pathogens [25], [30]. The fungal species used for testing PCR specificity included two other *Aspergillus* species (*A. flavus* and *A. terreus*), two other mold species (*F. solani* and *F. oxysporum*), several *Candida* and other common yeast species causing infections in humans such as *C. albicans, C. parapsilosis*, *C. dubliniensis, C. glabrata*, *C. tropicalis*, *C. neoformans*, *T. asahii, T. mucoides* and *T. inkin*. PCR amplification was carried out in a total volume of 50 µl containing 1× AmpliTaq PCR buffer I, 1 U AmpliTaq DNA polymerase (Applied Biosystems), 8 pmol each of AFUF and AFUR primers, 2 µl of DNA extracted from culture or other specimens and 0.1 mM of each dNTP. The PCR cycling conditions included one cycle of 95°C for 5 min, 55 cycles (30 cycles for DNA extracted from culture) of 95°C for 1 min, 65°C for 30 seconds and 72°C for 1 min and a final extension step at 72°C for 10 min. The amplicons were detected by 2% agarose gels [31]. Standard precautions were followed to avoid amplicon carry-over and false-positive PCR results as described previously [32]. The genomic DNA isolated from *A. fumigatus* was used as a positive control for PCR while water in place of DNA was used to serve as a negative control of PCR. To avoid false negative PCR results due to presence of PCR inhibitors, the presence of rat DNA in serum and BAL samples was confirmed by amplification of 18S rRNA gene with primers 18SF (5′-CTCTTAGCTGAGTGTCCCGC-3′) and 18SR (5′-CTTAATCATGGCCTCAGTTCCGA-3′) and by using the same protocol as described above, except that only 35 cycles of PCR amplification were performed.

### Detection of galactomannan (GM) and β-D-glucan (BDG) in serum and BAL specimens

The detection of GM was performed with Platelia *Aspergillus* EIA kit (Bio-Rad) according to the instructions in the supplier's manual and as described previously [17], [18]. Briefly, 300 µl of the serum or BAL specimen was mixed with 100 µl of 4% EDTA treatment solution and boiled for 3 min. After centrifugation at 10,000×g for 10 min, 50 µl of the supernatant and 50 µl of a reaction mixture containing peroxidase-conjugated anti-galactomannan monoclonal antibody EB-A2 were placed in the wells of a microtiter plate previously coated with the monoclonal antibody EB-A2 and incubated at 37°C. After 90 min, the plates were washed five times with washing solution and then 200 µl of TMB chromogenic substrate buffer was added. The plates were incubated for 30 min in dark at RT, and then 100 µl of 1.5 N sulfuric acid was added to stop the reaction. The optical density of each well was read at 450 nm with reference filter of 620 nm using a plate reader (Tecan, Model A-5082). The galactomannan levels were determined using a threshold index (of sample of threshold serum) and an index value >0.5 was considered as a positive result. The detection of BDG was performed by using the Fungitel kit (Associates of Cape Cod Inc., East Falmouth, MA, USA) against a purified Pachyman standard, which included a five-point two-fold curve ranging from 31 pg/ml to 500 pg/ml according to the instructions supplied by the manufacturer and as described previously [17], [18]. Briefly, serum or BAL samples (5 µl) were pretreated (in duplicate) for 10 min at 37°C with 20 µl of an alkaline reagent containing 0.6 M KCl and 0.125 M KOH. The BDG was then assayed with the Fungitell reagent in a kinetic, chromogenic format for 40 min at 37°C using BioTek plate reader (BioTek Instruments Inc., Winooski, VT, USA) with Gen5 software onboard to accomplish kinetic analysis of the microtitre plate at 405 nm. The concentration of BDG in each sample was calculated by using a calibration curve with standard BDG solutions. The samples, yielding absorbance values outside the range of the standard curve (>500 pg/ml), were diluted in reagent grade water and the test was performed again. A sample yielding BDG levels ≥80 pg/ml was taken as positive according to the instruction sheet provided with the kit.

### Statistical analyses

Categorical variables were expressed as number and/or percent. Statistical analysis was performed using chi-square test or Fisher's exact test as appropriate and probability levels less than 0.05 by the two-tailed test were considered as significant. The trend for positivity in serum samples at different time points was determined by chi-square test using the Cochrane-Armitage set for linear trend. The strength of agreement between the positivity for DNA in serum and BAL samples as well as the positivity for DNA and GM detection in serum was measured by robust kappa (κ) statistics. A κ coefficient of >0.6 indicated good agreement whereas a κ value of >0.8 signaled very good agreement. Statistical analyses were performed using SPSS 14.0 software for Windows (SPSS Inc., Chicago, IL, USA) and/or WinPepi software ver. 3.8 (PEPI for Windows, Microsoft Inc., Redmond, WA, USA).

## Results

### Development of experimental rat model for pulmonary aspergillosis

An inhalation chamber was designed for developing a murine model of invasive pulmonary aspergillosis through respiratory route as described in Materials and Methods. The exposure time of 5, 10, 15 or 20 min. were initially planned. The infecting dose was standardized by manually exposing four healthy rats in each group to *A. fumigatus* conidia in the inhalation chamber, the exposed rats were sacrificed immediately and the lungs were removed for viable count. An exposure time of 5 min yielded approximately 2×10^5^ cfu/g of lung tissue (ranging from 1.0×10^5^ to 2.5×10^5^ cfu/g of lung tissue among the four rats) in two separate experiments and was found appropriate for the development of IPA. Longer exposure times were not studied for determining the infecting dose.

Cortisone acetate was chosen as the single immunosuppressing agent and the dosing and frequency of cortisone acetate administration were adopted from previously published experimental rat models of pulmonary aspergillosis [26], [27]. The immunosuppression protocol with cortisone acetate (200 mg/kg body weight) given intramuscularly on Day -4, -2, Day 0 and Day 2 and Day 4 postinfection [26], [27] was tested for its efficacy in our studies. The total intended follow-up period of one week was based on recent observations that the initial 4 days of IPA are critical for accurate diagnosis and effective antifungal therapy in both, experimental models and human subjects [28], [29]. The lung homogenate from all 4 rats exposed for 5 min to *A. fumigatus* conidia and sacrificed on Day 2, 4 or 6 postinfection showed *A. fumigatus* hyphae in KOH-calcofluor mounts and also yielded the fungus in culture without causing mortality to allow follow-up of all the exposed animals while longer exposures resulted in animal mortality. On the basis of the above results (presence of fungal elements in microscopic examination of the lung tissue and positive lung tissue culture), the above immunosuppression schedule and the exposure time of 5 min was followed for the development of pulmonary aspergillosis in rats for the rest of the experiments.

### Development of a single-step PCR assay for detection of *A. fumigatus* DNA

A sensitive and specific single-step PCR amplification assay was developed for detection of *A. fumigatus* DNA by designing two (AFUF and AFUR) primers which target multi-copy ITS-1 region within rDNA and yield a small (∼174 bp) amplicon. The specificity of the primers for *A. fumigatus* was strongly suggested by BLAST searches as 100% identity was observed only with the ITS-1 region sequences from *A. fumigatus* but not from other *Aspergillus* species or other mold and yeast species and was confirmed experimentally. PCR amplification performed with DNA from reference strains of *A. fumigatus, A. terreus*, *A. flavus* and other mold or yeast species, yielded a single amplicon of ∼174 bp only from *A. fumigatus* but not from the other *Aspergillus* or other mold or yeast species. The lower limit of detection was 1 pg of *A. fumigatus* DNA which is roughly equivalent to 25 *A. fumigatus* genome copies (40 fg of genomic DNA per haploid genome). Thus, the analytical sensitivity of this assay was nearly same as that reported previously (>10 *A. fumigatus* cells) for the nested PCR assay [25] but does not involve the risk of amplicon carry-over and cross-contamination of samples due to opening of first round PCR tubes for an aliquot for second round amplification. To avoid false-negative results due to presence of inhibitors of PCR in serum/BAL samples, a PCR assay using a primer pair targeting the murine 18S rRNA gene was also developed. This control PCR assay successfully amplified a DNA fragment of the expected size (156 bp) with DNA isolated from rat serum.

### Levels of Glucan and galactomannan and detection of *A. fumigatus* DNA in normal rats

The culture of lung tissue and BAL samples of all the 12 control rats were uniformly negative for *A. fumigatus* or any other fungal pathogen. None of the serum sample from control rats yielded BDG (mean value  = 16.63+8.54 pg/ml) or GM (mean value  = 0.15+0.036 pg/ml) levels above the cut-off values or showed the presence of *A. fumigatus* DNA by PCR. Similarly, none of the BAL sample from control rats yielded BDG (mean value  = 30.88+17.80 pg/ml) or GM (mean value  = 0.26+0.06 pg/ml) levels above the cut-off values or showed the presence of *A. fumigatus* DNA by PCR, as expected.

### Microscopy and culture of lung tissues of rats exposed to *A. fumigatus* conidia

**Figure 2:**
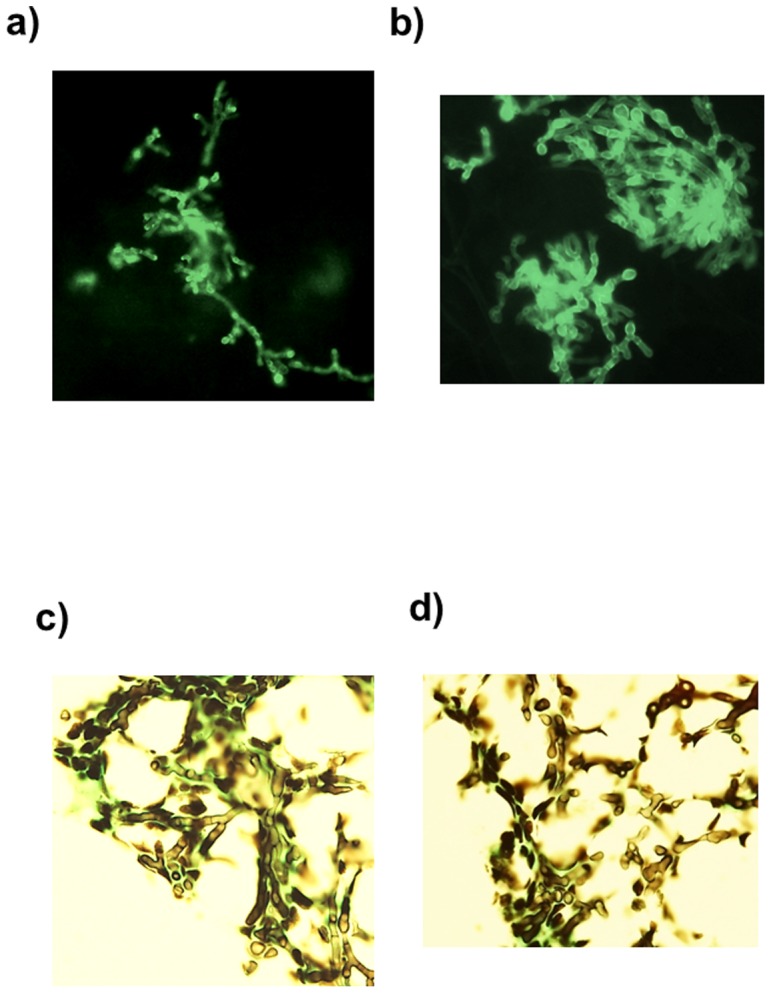
KOH-calcoflour mounts and histopathology of lung tissue sections. The KOH-calcoflour mounts (**a** and **b**), and histopathology (**c** and **d**) of lung tissue sections obtained from 4 immunosuppressed rats sacrificed on Day 3 postinfection showing abundant growth of *A. fumigatus*.

The results of microscopic examination by KOH-calcofluor mount and culture of 30 rats sacrificed in groups of six rats each on Day 1, 3, 4, 5, and 7 are presented in Table 1. The cultures of lung tissue of all the rats sacrificed on Day 1, Day 3, Day 4, and Day 5 were positive, while culture from 4 of 6 (67%) animals sacrificed on Day 7 were positive for *A. fumigatus*. Overall, 28 of 30 (93%) lung tissue specimens were positive for the presence of *A. fumigatus* by culture. The microscopic examination of lung tissue by KOH-calcofluor mount showed the presence of the fungus in all the animals sacrificed on Day 3 and Day 4 and nearly all the animals (5 of 6, 83%) sacrificed on Day 5 (data from four of six animals sacrificed on Day 3 are shown in Figure 2). However, only 2 of 6 (33%) and 3 of 6 (50%) specimens collected on Day 1 and Day 7, respectively, showed the presence of mold in lung tissue (Table 1). Thus, 22 of 30 (73%) lung tissue specimens from experimental animals were positive for the presence of fungal elements (*A. fumigatus*) by direct microscopy. Interestingly, there was good agreement between the decreasing trend for the presence of fungus in lung tissue by microscopy and lung tissue culture positivity for *A. fumigatus* in animals sacrificed on Day 7, reflecting clearing of infection due to waning of immunosuppression.

**Table 1:** Comparative results of culture and KOH-calcofluor microscopy of lung tissue samples from *A. fumigatus*-infected rats.

No. of days postinfection	No. of rats in group	No. (%) of samples positive for *A. fumigatus* by	
		Culture	KOH-calcofluor microscopy
1	6	6 (100)	2 (33)
3	6	6 (100)	6 (100)
4	6	6 (100)	6 (100)
5	6	6 (100)	5 (83)
7	6	4 (67)	3 (50)*
Overall		28 (93)	22 (73)

*Kappa (κ)  = 0.67 (no value could be obtained for κ for other time points since all lung tissue samples were positive for culture).

### Detection of BDG, GM and *A. fumigatus* DNA in serum and BAL specimens

Serum and BAL specimens tested in duplicate from all the infected animals were positive (≥80 pg/ml) for BDG (Table 2). The individual values varied from 96.4 pg/ml to 626 pg/ml. For GM detection, 24 of 30 (80%) serum samples while all 30 (100%) BAL specimens yielded values above the cut-off index value of >0.5 (Table 2). The index values for serum and BAL samples ranged from 0.23 to 5.21. The positivity for GM detection in serum specimens was highest (6 of 6, 100%) on Day 3 and Day 4, and lowest (3 of 6, 50%) on Day 7 (Table 2). The positivity for the detection of *A. fumigatus* DNA from infected animals was lower (16 of 30, 53%) in serum specimens but much higher (27 of 30, 90%) in BAL samples. The positivity for *A. fumigatus* DNA in serum specimens was highest (5 of 6, 83%) on Day 4 and lowest (1 of 6, 17%) on Day 1 (Table 2). Interestingly, the combined BDG, GM and *A. fumigatus* positivity in serum samples was highest on Day 3 (16 of 18 positive) and Day 4 (17 of 18 positive) but declined on Day 5 (13 of 18 positive) and Day 7 (12 of 18 positive) with waning of immunosuppression and the difference between Day 4 and Day 7 was nearly statistically significant (p = 0.088). Excluding the data for Day 1 (to allow for germination of conidia and growth of *A. fumigatus* in the lungs for seeding blood), linear trend for decline in positivity for the three markers in serum for Day 3, Day 4, Day 5 and Day 7 was statistically significant (p = 0.033). Also, the positivity for both GM and DNA in serum samples declined on Day 5 and Day 7 postinfection and there was a trend towards an agreement (albeit a low agreement) between detection of GM versus DNA in serum samples, particularly on Day 7 reflecting clearing of infection due to waning of immunosuppression. On the other hand, positivity for *A. fumigatus* DNA in BAL specimens was highest (6 of 6, 100%) on Day 1, Day 4 and Day 5 and lowest (4 of 6, 67%) on Day 7 (Table 2). Lack of detection of *A. fumigatus* DNA in the remaining serum and BAL samples was not due to the presence of PCR inhibitory substances in DNA preparations as all samples readily amplified rat 18S rRNA gene sequence. There was also good agreement among rats for detection of *A. fumigatus* DNA in serum versus BAL samples on Day 7 and nearly good agreement on Day 3 postinfection while the agreement on other days could not be calculated since all six BAL samples from animals sacrificed on Day 1, Day 4 and Day 5 tested positive for *A. fumigatus* DNA (Table 2). The BAL specimens from infected animals were also cultured and 3 of 6 (50%), 2 of 6 (33%) and 2 of 6 (33%) BAL specimens on Day 1, Day 3, and Day 5 postinfection, respectively, yielded *A. fumigatus* (Table 2).

**Table 2:** Positivity for the detection of 1,3 β-D glucan (BDG), galactomannan (GM) and *A. fumigatus* DNA in serum and bronchoalveolar lavage (BAL) samples and culture positivity in BAL of experimentally infected rats sacrificed on different days postinfection.

No. of days post-infection	No. of rats in group	No. (%) of sera positive for				No. (%) of BAL samples positive for				Kappa (κ)^b^
		BDG	GM	DNA	Kappa (κ)^a^	BDG	GM	DNA	Culture	
1	6	6 (100)	5 (83)	1 (17)	−0.38	6 (100)	6 (100)	6 (100)	3 (50)	*
3	6	6 (100)	6 (100)	4 (67)	*	6 (100)	6 (100)	5 (83)	2 (34)	0.57
4	6	6 (100)	6 (100)	5 (83)	*	6 (100)	6 (100)	6 (100)	ND	*
5	6	6 (100)	4 (67)	3 (50)	0.0	6 (100)	6 (100)	6 (100)	2 (34)	*
7	6	6 (100)	3 (50)	3 (50)	0.33	6 (100)	6 (100)	4 (67)	0 (0)	0.67
Overall		30 (100)	24 (80)	16 (53)		30 (100)	30 (100)	27 (90)	7 (23)	

a Kappa (κ) calculated for agreement between GM versus DNA detection in serum samples.

b Kappa (κ) calculated for agreement between DNA detection in serum samples versus DNA detection in BAL samples.

*No value could be obtained for kappa (κ) since all samples tested for GM or DNA in one group were positive.

ND, not done.

The per cent of positive tests for BDG, GM, and *A. fumigatus* DNA in serum samples of infected rats on day 1, 3, 4, 5, and 7 was 100, 83 and 17; 100, 100 and 67; 100, 100 and 83; 100, 67 and 50; and 100, 50 and 50, respectively (Table 2). The per cent of positive tests for BDG, GM, and *A. fumigatus* DNA in BAL samples of infected rats on day 1, 3, 4, 5, and 7 was 100, 100 and 100; 100, 100, and 83; 100, 100 and 100; 100, 100 and 100; and 100, 100 and 67, respectively (Table 2).

The detection of BDG and GM in serum samples was also compared with the detection of *A. fumigatus* DNA. Of the 30 BDG-positive serum samples, only 16 (53%) were also positive for *A. fumigatus* DNA. Although per cent positivity for *A. fumigatus* DNA in serum samples positive for GM was nearly same (13 of 24, 54%) as reported above among BDG-positive serum samples, however, 3 of 6 serum samples negative for GM were positive for *A. fumigatus* DNA while serum samples of 3 rats were negative for both, GM and *A. fumigatus* DNA.

**Figure 3:**
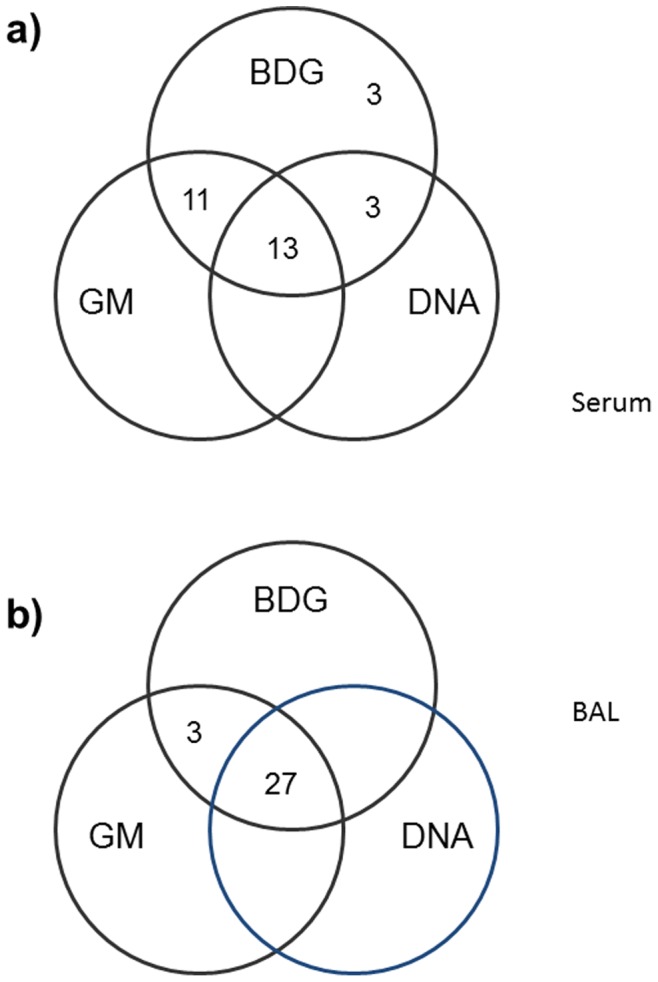
Venn diagrams showing combined data for BDG, GM and *A. fumigatus* DNA in serum and BAL samples. The Venn diagrams show the number of serum (**a**) and BAL (**b**) samples positive individually and in various combinations for BDG, GM and *A. fumigatus* DNA from experimental rats.

Collectively, serum samples from 13 (43%) experimental rats were positive for all the three biomarkers (BDG, GM and *A. fumigatus* DNA) (Figure 3a). Additionally, 14 (47%) serum samples were positive for two markers. These included 11 serum samples that were positive for BDG and GM and 3 serum samples positive for BDG and DNA. Three serum samples were positive for BDG only (Figure 3a). On the other hand, BAL samples from 27 experimental rats were positive for all the three biomarkers (Figure 3b). The remaining 3 BAL samples were positive for two diagnostic markers (GM and BDG) (Figure 3b). Seven of 24 BAL samples also yielded the fungus in culture.

## Discussion

Timely diagnosis of IPA remains a major challenge for clinical microbiology laboratories. Experimental models of IPA have been developed in rabbits, guinea pigs and rats and the oral route (endotracheal instillation or aerosol inhalation) of infection is preferred over intravenous inoculation as it resembles more closely with the infection in humans. The efficacy of various biomarkers has been evaluated either alone or in combination to improve the diagnosis of IPA [22], [23], [28], [33], [34]. Recent studies have shown that the initial 4 days of IPA are critical for accurate diagnosis in both, experimental models and human subjects [28], [29]. Furthermore, the reduction in fungal burden in an experimental model of IPA was recently shown to be negligible when antifungal therapy was delayed by ≥72 hours, emphasizing the value of early diagnosis in proper disease management [28]. Based on these observations, the maximum follow-up period of one week was chosen in this study.

We preferred rats over rabbits or guinea pigs since this animal is more readily available and provides sufficient amount of lung tissue and blood samples per animal to investigate diagnostic aspects of IPA at a lower cost. We also chose the aerosol inhalational model as endotracheal instillation is cumbersome requiring general anesthesia [23], [33]. Microscopic examination of lung tissue samples from exposed animals with KOH-calcofluor mount showed the presence of fungal hyphae providing histological evidence of infection as demonstrated in several other studies involving both, endotracheal instillation and aerosol inhaltion models of IPA in rabbits and guinea pigs [28], [33], [34]. Some investigators using endotracheal instillation or aerosol inhalation models of experimental IPA in rats, rabbits and guinea pigs have used more severe immunosuppressive regimens (cyclophosphamide + cortisone acetate) or exposed animals to higher fungal load for longer duration that produced more lethal infection with animals dying within 5 days postinfection [28], [34]–[36]. Early mortality of animals hampers efforts to study the comparative performance and kinetics of different biomarkers during the entire period of follow-up. We used a single immunosuppressive agent (cortisone acetate) and also standardized the inoculum and time of exposure in the acrylic chamber to ensure that the exposed animals are infected but survive during the entire intended period of follow-up (7 days).

We also developed a single-step, highly sensitive and *A. fumigatus*-specific PCR assay by re-designing nested primers used previously to minimize amplicon carry-over and cross-contamination of samples so as to avoid false-positive results [25]. Consistent with BLAST predictions, PCR amplification yielded an expected (174 bp) amplicon from *A. fumigatus* only and not from other *Aspergillus* spp. or other mold and yeast species with a sensitivity comparable to nested PCR assays [25], [37], [38]. Real-time PCR (rt-PCR) assays, however, are more sensitive but they require expensive equipment and probe primers [22], [23], [33], [34].

None of the exposed animals died during the course of this study indicating low-dose infection. Histological evaluation showed that the experimental model successfully established lung infection with *A. fumigatus*. The KOH-clacofluor microscopy results showing the presence of fungus in fewer animals (2 of 6, 33%) on Day 1 than on Day 3 and Day 4 postinfection (12 of 12, 100%) indicate that the fungal load in lung tissue increased with the progression of the disease. This is consistent with previous reports where most of the experimental animals (rabbits or guinea pigs) infected by endotracheal instillation or aerosol inhalation and sacrificed within 24 h of exposure mostly showed conidia and limited pathological changes [28], [34], [35]. High lung tissue culture positivity was also reported up to Day 4 in the rat aerosol inhalation model of IPA [22]. The positivity for lung tissue cultures and KOH-calcofluor microscopy on Day 7 decreased considerably, possibly due to waning of the effects of immunosuppression and there was good agreement between the declining positivity for the presence of fungus in the lung by microscopy versus lung tissue culture positivity. In other studies using endotracheal instillation or aerosol inhalation but more severe and prolonged immunosuppression, the lung tissue cultures remained positive for longer duration. However, the fungal burden still showed a declining trend over time [28], [34], [36]. The culture positivity from BAL samples in our study was lower (7 of 24, 29%) compared to lung tissue culture (28 of 30, 93%) and it also showed a decreasing trend with time. Other investigators using experimental models of IPA in rats or rabbits with endotracheal instillation or aerosol inhalation have reported slightly higher (40–50%) BAL culture positivity, possibly due to more severe immunosuppression or due to higher infecting dose [22], [23], [28]. However, BAL culture positivity in these studies also showed a declining trend over time [22], [28].

Similar to other recent studies, the specificity of the three biomarkers for diagnosis of IPA was 100% as none of the serum or BAL specimens of 12 control rats yielded positive results for BDG, GM or *A. fumigatus* DNA [22], [23], [34]. BDG was uniformly detected in all serum and BAL specimens, however, detection of GM and *A. fumigatus* DNA in BAL samples was superior to their detection in serum samples with GM exhibiting higher sensitivity than *A. fumigatus* DNA in both, serum and BAL specimens. BAL fluid testing for GM has also been shown to increase diagnostic performance over that of blood testing in patients with IPA [39]–[41]. Collectively, only 14 of 30 (47%) serum samples but 27 of 30 (90%) BAL specimens were positive for all the three biomarkers. Only one study with oral route (endotracheal instillation) of infection in rats (immunosuppressed consecutively with cyclophosphamide and prednisolone administered only until the day of inoculation) has detected all three biomarkers, however, direct comparisons with our data are difficult to make since only serum samples were analyzed in that study and only 2 animals each were followed for 4 and 5 days [42]. Nonetheless, only 55%, 55% and 67% of serum samples during the first three days were positive for GM, BDG and *A. fumigatus* DNA with only 6 of 18 (33%) samples yielding positive results for all the three biomarkers [42]. In our study, the highest positivity for the three biomarkers in serum samples was obtained on Day 3 and Day 4 at the peak of immunosuppression which is also likely to correlate best with humans that are constantly immunosuppressed. This was also evident from a nearly good agreement for the decline in positivity for GM and DNA in serum samples and a good agreement for the decline in positivity for detection of *A. fumigatus* DNA in serum versus BAL samples on Day 7 postinfection indicating that the infection was established in the lungs but was contained by the host and was subsequently cleared due to waning of immunosuppression.

Other studies using respiratory route (endotracheal instillation or aerosol inhalation) of infection in experimental animal models of IPA have usually compared the performance of two biomarkers; typically GM and PCR or BDG in BAL and/or blood/serum samples [22], [23], [28], [33], [34], [43]. Earlier studies showed that GM detection in BAL was more sensitive than DNA detection [33], [43]. Recent studies have shown that DNA detection by rt-PCR is more sensitive than GM detection in BAL [23] and serum samples [34]. Conflicting results have also been reported from patients with IPA. While GM detection was less sensitive than DNA detection by pan-*Aspergillus* rt-PCR in both, BAL and serum samples [15], [44], GM detection in BAL was superior when DNA was detected by *A. fumigatus*-specific rt-PCR assay [44]. Two recent systematic reviews have also concluded that PCR is as sensitive as GM in BAL samples and both tests provide optimal sensitivity with no loss of specificity [19], [45].

A positive trend was observed between BDG and GM values in serum samples. Other studies in experimental animal models have also shown that BDG and GM have similar kinetics during infection and detection of both the markers is helpful in early diagnosis of IPA [24], [25], [28]. Application of both the tests is also preferred in human subjects for the diagnosis of IPA since BDG demonstrates high negative predictive value while two consecutive serum samples showing GM positivity provide strong evidence for the diagnosis of IPA in human subjects [11]. Combined detection also eliminates false positive or discrepant reactions occurring with either test alone but increases the cost [46]. It is, therefore, preferable to combine GM detection with DNA by PCR or rt-PCR for economic reasons since BDG and GM exhibit similar kinetics and BDG, being a panfungal marker, may not contribute significantly to enhance the specificity of the test. Furthermore, BDG has a low positive predictive value for the diagnosis of IPA [11]. Our data also show that combined detection of both GM and DNA in BAL and detection of either GM or DNA in serum has a high (90%) sensitivity and is sufficient for the detection of IPA in vast majority of infected animals. More importantly, high sensitivity for detection of GM and DNA in BAL samples from exposed animals during early stages of infection (within three days) suggests that rapid diagnosis of IPA is feasible by detecting these two biomarkers.

Our study has few limitations. Only 6 animals were used in each group which limited accurate statistical analyses of positive results for the three biomarkers in serum and BAL samples. We also did not perform quantitative cultures of the lung tissue from infected animals to ascertain fungal burden at different time points and other organs were not studied for the presence of *A. fumigatus* to determine if infection from the lungs had spread to any other organ.

## Conclusions

A sensitive and specific, single-step PCR assay has been developed for the detection of *A. fumigatus* DNA in serum and BAL specimens for the diagnosis of IPA. Biomarker detection in BAL samples from 27 of 30 (90%) rats was positive and superior to serum (13 of 30, 43%) for all the three biomarkers (BDG, GM and *A. fumigatus* DNA) for the diagnosis of IPA. However, combined detection of GM and DNA in BAL and detection of either GM or DNA in serum was also positive in 27 of 30 (90%) animals. The highest positivity for the three biomarkers in serum samples from infected rats was obtained on Day 3 and Day 4 representing the peak of immunosuppression which is also likely to correlate best with humans that are constantly immunosuppressed. This is encouraging since BAL collection is more invasive than obtaining blood and thus may not always be available. Considering the low positive predictive value of BDG, similarities of experimental animal model with human infection and economic use of resources, the detection of GM and/or DNA in serum and BAL samples has the potential to be used in overall diagnostic strategy in high-risk patients.
